# Compounds from Silicones Alter Enzyme Activity in Curing Barnacle Glue and Model Enzymes

**DOI:** 10.1371/journal.pone.0016487

**Published:** 2011-02-17

**Authors:** Daniel Rittschof, Beatriz Orihuela, Tilmann Harder, Shane Stafslien, Bret Chisholm, Gary H. Dickinson

**Affiliations:** 1 MSC Division, Duke University Marine Laboratory, Nicholas School of the Environment, Beaufort, North Carolina, United States of America; 2 Centre for Marine Bio-Innovation, University of New South Wales, Sydney, New South Wales, Australia; 3 Center for Nanoscale Science and Engineering, North Dakota State University, Fargo, North Dakota, United States of America; University of Crete, Greece

## Abstract

**Background:**

Attachment strength of fouling organisms on silicone coatings is low. We hypothesized that low attachment strength on silicones is, in part, due to the interaction of surface available components with natural glues. Components could alter curing of glues through bulk changes or specifically through altered enzyme activity.

**Methodology/Principal Findings:**

GC-MS analysis of silicone coatings showed surface-available siloxanes when the coatings were gently rubbed with a cotton swab for 15 seconds or given a 30 second rinse with methanol. Mixtures of compounds were found on 2 commercial and 8 model silicone coatings. The hypothesis that silicone components alter glue curing enzymes was tested with curing barnacle glue and with commercial enzymes. In our model, barnacle glue curing involves trypsin-like serine protease(s), which activate enzymes and structural proteins, and a transglutaminase which cross-links glue proteins. Transglutaminase activity was significantly altered upon exposure of curing glue from individual barnacles to silicone eluates. Activity of purified trypsin and, to a greater extent, transglutaminase was significantly altered by relevant concentrations of silicone polymer constituents.

**Conclusions/Significance:**

Surface-associated silicone compounds can disrupt glue curing and alter enzyme properties. Altered curing of natural glues has potential in fouling management.

## Introduction

For the management of biological fouling, foul-release coatings are an alternative to broad spectrum biocides. Weak attachment of organisms on foul-release surfaces facilitates cleaning. For all but continuous use and high speed ships, periodic grooming or cleaning is required to maintain performance [1]. Existing commercial foul release coatings are based upon silicone polymers.

Weak attachment on silicone foul-release coatings is attributed to a combination of physical and chemical properties of the polymer. Physical properties include elastic modulus, coating thickness, and Baier's “bioadhesive minimum” or “*theta surface*” (critical surface tension, a property of surface energy, between 20–27 mN m^−1^)[2]–[6], while chemical properties may include catalysts (e.g. organotins, organobismuths, etc.), silicone oils, and free silicone components that migrate to the surface of the polymer [7]–[9]. Surface-associated components of silicone coatings have the potential to interfere with cross-linking of biological glues [9]. Silicon is incorporated into the adhesive plaque of barnacles grown on silicone coatings, suggesting release and uptake of uncross-linked PDMS [7], [8]. Biochemical mechanisms that might alter adhesive curing are the focus of this report.

At the biochemical level, natural marine glues are complex, multicomponent systems [10]. Marine glues displace water, form bonds with the substrate, and are stabilized by cross-linking [11]. Enzymes and/or specific cofactors such as metal ions are essential to curing [12]–[15]. Disruption of this complex assembly alters glue properties [15]–[17].

Potential mechanisms for altering glue properties include perturbation of: spatial and temporal activation of components, presentation of adhesive motifs, assembly, and enzymatic cross-linking of structural proteins. Alteration of curing enzyme activity, specifically and non-specifically, are potential mechanisms. Analogous to synthetic adhesives, we hypothesize that natural glues are sensitive to catalyst activity levels. Hence, we suspect that compounds associated with silicones can alter enzyme activity, glue curing and glue properties.

Barnacles are a major target of fouling management. At the biochemical level, barnacle glue curing has similarities to blood clotting [15]. Curing involves proteolytic activation of enzymes and structural precursors, transglutaminase cross-linking, and assembly of fibrous proteins. Proteolytic activation of structural proteins maximizes the potential for bonding interactions with other proteins and with the surface. Domains exhibiting compatible adhesive motifs [18]–[20] become available to present to surfaces following activation. Transglutaminase [15] and other kinds of cross-linking [12], [21], [22] stabilize the glue. Thus, adhesion and curing involve at least two enzymatic steps. Because the curing involves rearrangement of structural proteins and cross-linking of the proteins for stability, alteration of enzyme activities could change the properties of the glue.

We tested the hypothesis that compounds associated with silicone polymer surfaces alter the activity of enzymes that participate in glue curing. Three sets of experiments were conducted. First, coupled gas chromatography-mass spectrometry was used to determine the presence of compounds on silicone surfaces. Next, we determined if surface-associated compounds altered trypsin-like serine protease and transglutaminase activity in curing barnacle glue. Finally, changes in the activities of commercial trypsin and transglutaminase were quantified in the presence of: A) 30 second methanol rinses of silicone surfaces and compounds transferred to cotton swabs during gentle rubbing of polymer surfaces; and B) individual components of silicone polymers alone and in combination. Results show that compounds associated with silicone surfaces alter transglutaminase activity in curing glue and trypsin and transglutaminase activity in purified enzymes.

## Materials and Methods

### Methanol rinses of commercial silicone films: GC-MS

Dow Corning Silastic T2^®^ and International Veridian^®^ were analyzed using coupled gas chromatography-mass spectrometry. These two silicones were chosen since they are used experimentally and were readily available in a leached state. Silicones were prepared on 7.6×15.2×0.64 cm glass panels as described by Holm et al. [23]. After preparation, T2^®^ and Veridian^®^ coated panels were conditioned in flowing seawater until barnacles would settle on the surfaces in the laboratory. Soaking for 5 days (T2^®^) and 35 days (Veridian^®^) respectively was required. After this, the panels were used as barnacle growth substrates for approximately 2½ years. After this 2½ year interval, the panels were used in this study.

Chemical analyses were conducted at the Institute for Chemistry and Biology of the Marine Environment (ICBM; Oldenburg, Germany). A 30 second, 30 µl methanol rinse was used to obtain compounds from the silicone surfaces. Eluates were analyzed undiluted by coupled gas chromatography-mass spectrometry. A WCOT VF-5ms capillary column (Varian, USA) (30 m×0.25 mm×0.25 µm film thickness) was mounted on a Varian 3900 gas chromatograph equipped with a Saturn 2100 T (Varian, USA) ion trap mass selective detector. Samples were injected in splitless mode with an inlet pressure of 72 kPa. The injection port and the interface were held at 260°C. The gas chromatograph was held at 70°C for 1 min and ramped at 15°C min^−1^ to 150°C, 20°C min^−1^ to 250°C and held at this temperature for 2 min. Finally, the column was cleaned at 320°C for 1 min. Helium was used as the carrier gas. The mass selective detector was operated in scan mode (m/z 10–650). The electron impact ion-spectra of silicone eluate components were compared with entries in the NIST mass spectral library (NIST V. 2005). To account for background organosiloxane contaminants, blank samples of methanol were run after every 5 analyses to identify and subtract system specific siloxane peaks.

### Methanol rinses of commercial silicone films: enzyme assays

In addition to T2^®^ and Veridian^®^ silicone we included RTV-11^®^, a commercial silicone used experimentally, and Intersleek 425^®^, a commercial foul-release coating, in our enzyme analyses. Intersleek^®^ and RTV-11^®^ silicones were prepared as draw downs on epoxy-primed 4×8 inch (10.2×20.3 cm) marine grade aluminum panels (Q-panel, USA), and conditioned in flowing seawater for 7 days after preparation. Intersleek^®^ and RTV^®^ silicones were then used intermittently as barnacle growth substrates and for barnacle reattachment studies, and were immersed in seawater for a period of approximately 11 months. After this 11-month interval, panels were used in this study.

For enzyme assays, silicone substrates were rinsed thoroughly with deionized water and dried in air prior to methanol rinses. A 30 second, 60 µl methanol rinse was used to obtain compounds from the silicone surfaces. Ten 60 µl rinses were recovered with a glass Pasteur pipette for each silicone, combined in a glass test tube, and air dried in a fume hood. The residual was resuspended in 10 µl 100% methanol for the assays described in this section. Residues were agitated gently to aid redissolution. Two controls were implemented to ensure that the 10 µl HPLC grade methanol used to resuspend residuals did not impact enzymatic activity. First, for all assays, 10 µl methanol was incubated with enzyme substrate. Absorbance values from these samples served as the basis for statistical comparisons. Second, a set of trypsin and transglutaminase assays were carried out with curing barnacle glue, with one set of samples containing 10 µl 100% methanol, and a second set with methanol replaced by 10 µl deionized water. These assays were run as described below, with the exception of 3 µl curing glue used in trypsin assays. Assays were run in a paired design, with glue from the same barnacle used for the methanol and the deionized water sample.

Rearing of barnacles *Amphibalanus* ( = *Balanus*) *amphitrite* [24] and collection of curing glue were conducted as described in Dickinson et al. [15]. Trypsin and transglutaminase were assayed with enzyme specific substrates. Enzyme assays were conducted with curing barnacle glue, and with commercially available purified enzymes. Assays with curing glue enabled determination of variability among individual barnacles. Assays with purified enzymes provided precise control of reaction conditions, including concentration of the enzyme, substrate, and cofactors.

Trypsin assays were conducted using a BAPNA (Nα-benzoyl-DL-arginine 4-nitroanilide; Acros Organics #227740010) substrate. BAPNA was prepared at 0.044% (w/v) by first dissolving BAPNA in DMSO (dimethylsulfoxide: 1% v/v) and then adding 50 mM Tris buffer, pH 8.0. Reaction conditions (pH, incubation temperature, buffer concentration) followed Dougherty [25] who optimized reaction conditions for general protease activity in curing glue from the barnacle *Chthamalus fragilis*.

Trypsin assays with curing barnacle glue were conducted by first adding 800 µl BAPNA solution directly to test tubes containing surface eluate resuspended in 10 µl methanol, or 10 µl methanol only (control). 6 µl unpolymerized glue was added to each tube, vortexed, and incubated at 37°C for 1 hr. Each silicone and the methanol control was tested with glue from the same barnacle. Following incubation, all samples were centrifuged at 9000 rpm for 10 min in a Fisher Scientific MicroD centrifuge and then placed on ice. Samples were transferred to a quartz semi-micro cuvette (Starna Cells #9-Q-10) and optical density at 405 nm (OD_405_), referenced to Tris buffer alone, was read on a Hewlett Packard 8451A diode array spectrophotometer. Samples were staggered in ∼8 sample groups (each with controls) so that all samples could be read within 10 min of centrifugation.

Assays with purified trypsin were conducted in the same manner as described above, however, 6 µl of a purified trypsin solution was used in place of native glue. Bovine pancreatic trypsin was used (Sigma #T1426) and mixed to 44.68 BAEE units/ml for a total of 0.268 BAEE units per assay. This trypsin activity level was chosen to be measurable and within the range of values observed previously for barnacle glue [17]. Maximum trypsin activity reported was 38.25 BAEE units/ml with a mean value of 14.74 BAEE units/ml.

Transglutaminase activity assays were conducted using a transglutaminase assay kit (Sigma-Aldrich #CS1070), and followed the manufacturer's directions including those for use with an inhibitor. Assays were based on the reaction of transglutaminase with a cadaverine coated 96-well plate. For assays with native barnacle glue and with purified transglutaminase, 45 µl deionized water was added to test tubes containing silicone eluate resuspended in 10 µl methanol or 10 µl methanol only (control). The solutions were mixed gently and then transferred to cadaverine coated plate wells. For assays with native transglutaminase, 1 µl of unpolymerized glue was added to each well, whereas for assays with purified transglutaminase, 5 µl of a 20 milliunits ml^−1^ purified transglutaminase solution was added for a total of 0.1 milliunits used (transglutaminase from guinea pig liver, Sigma #T5398, was diluted from a 2 unit ml^−1^ stock solution which contained 10 mM DTT and 1 mM EDTA). As in trypsin assays, this activity level was chosen to be measurable and in the range of values observed previously for barnacle glue [17]. A 0.1 milliunits transglutaminase solution yielded an average OD_450_ value of 0.45, whereas the maximum OD_450_ for barnacle glue was 0.52 and mean OD_450_ was 0.28. Wells were mixed gently by tapping on the plate and incubated at room temperature for 3–5 min before adding assay buffer. At the completion of the assay, OD_450_ was read on a SpectraMax M2 plate reading spectrophotometer (Molecular Devices). For assays with curing glue, glue from the same individual barnacle was used to test each silicone and the methanol control.

### Mechanical removal of compounds on silicone films: GC-MS

After speaking with a scientist from the coatings industry regarding detection of surface-available compounds on silicone coatings, we changed techniques to eliminate exposure of the silicone film to methanol. A 2 cm^2^ silicone surface was rubbed gently for 15 seconds with a cotton swab and then the swab was eluted with methanol. We used eight model polysiloxane formulations described previously [26]. Each combination of the following variables was prepared: low or high molecular weight oligomers, low or high concentration of cross-linker and, with or without added silicone oil. The model polysiloxane coatings were prepared in 24 well polystyrene plates modified with epoxy-primed marine grade aluminum discs, as described previously [27]. These coatings were conditioned in deionized water for 14 days prior to their use in the assays described here.

Cotton swabs used to sorp compounds from model polysiloxanes were first cleaned by dripping approximately 1 ml of 100% methanol over the entire surface of the cotton swab until the methanol dripped off. Cotton swabs were then placed on clean aluminum foil and air dried overnight before use. Each silicone formulation was swabbed in triplicate with: 1) a cleaned and dried cotton swab; and 2) a cleaned cotton swab that had been pre-wetted with approximately 500 µl of 100% methanol. To swab silicones, the dry or wetted cotton swab was rubbed over the entire surface of the silicone-coated well by pressing down lightly with a circular motion for 15 seconds. For GC/Mass spec analysis the tip of the cotton swab was cut off and placed in a 1 ml borosilicate glass autosampler vial (Wheaton #223682) and sealed with parafilm. Before use, vials were cleaned with methanol and then baked in a muffle furnace at 500°C for 1 hr. Samples were sent overnight mail to the University of New South Wales for analysis.

For GC-MS analysis, cotton swabs were eluted by adding 1 ml of 100% methanol directly to the glass vials containing the cotton swab tips. Cotton swabs were left in methanol for 30 min. Elutions of dry swabs were concentrated 3 times by drying the eluate completely on a SpeedVac^®^ and then resuspending the residual in 330 µl methanol. Eluates of methanol wetted swabs were analyzed without concentration. Swabs were analyzed by coupled gas chromatography-mass spectrometry. A HP5-MS capillary column (Hewlett Packard, USA; 30 m×0.25 mm×0.25 µm) was mounted on an Agilent 6890N gas chromatograph interfaced to a HP5973N mass selective detector operated in electron impact mode at 70 eV. Samples were injected and run as described above.

### Mechanical removal of compounds on silicone films: enzyme assays

Enzyme assays were conducted on compounds from model polysiloxane coatings obtained by rubbing with cleaned and dried cotton swabs as described above. Wetted cotton swabs were not included in enzyme assays. Compounds sorped to the cotton swabs were eluted by placing it at the opening of a glass test tube and slowly dripping 100% methanol onto the swab until 200 µl ran off into the tube. The same procedure was used to generate controls from cleaned and dried cotton swabs. Enzyme assays with cotton swab eluates were conducted with purified enzymes only. All eluates were dried completely in a fume hood. Residues were resuspended directly in enzyme assay buffer. This change was made due to concern that addition of methanol might alter enzyme activity.

For trypsin assays, BAPNA (Nα-benzoyl-DL-arginine 4-nitroanilide) substrate was used and prepared at 0.044% (w/v) by first dissolving BAPNA in DMSO (dimethylsulfoxide: 1% v/v) and then adding HPLC water. A trypsin solution was made at 0.45 µg µl^−1^ in 1 mM HCl using bovine pancreatic trypsin (Sigma #T1426). This solution was then diluted to 3.0 µg ml^−1^ (42.9 BAEE units/ml) in seawater for use in assays and kept on ice at all times. Seawater served as the assay buffer. For assays, 90 µl of the 3.0 µg ml^−1^ trypsin solution (3.86 BAEE units total) was added directly to each test tube containing dried cotton swab eluates. Note that the trypsin activity level was higher than in previous assays to enhance sensitivity. Tubes were mixed and 80 µl from each tube was transferred to a multiwell plate. 170 µl BAPNA solution was added to each well, the plate was incubated at 37°C, and OD_405_ was read every 5 min for 15 min. Activity was calculated as µMoles substrate hydrolyzed/ml/min.

Transglutaminase activity assays were conducted using a transglutaminase assay kit (Sigma-Aldrich #CS1070), and generally followed the manufacturer's directions. In these assays, 50 µl of 2 milliunits ml^−1^ purified transglutaminase solution (0.1 milliunits total; diluted from a 2 unit ml^−1^ stock solution which contained 10 mM DTT and 1 mM EDTA) was added directly to each test tube containing dried cotton swab eluates. Tubes were mixed and 50 µl from each tube was transferred to cadaverine coated plate wells.

### Effects of silicone components on enzyme activities

Enzyme assays were conducted with five components of model polysiloxane coatings: silicone oil (viscosity 40–50 cSt: Gelest DES-T15); low molecular weight PDMS oligomers (700–800 cSt: Gelest DMS-S27); medium molecular weight PDMS oligomers (1000 cSt: Dow Corning 200 Fluid, Sample #321275); and high molecular weight PDMS oligomers (5000 cSt: Gelest DMS-S35). Purified trypsin and transglutaminase activity was tested with silicone components alone and in combination. In order to determine the quantity of each component to test in enzyme assays, the mass of silicone eluted from a model polysiloxane coating was empirically tested. Silicone made from low molecular weight oligomers, low cross-linker, with no oil, was swabbed with an unmodified dry cotton swab, and compounds taken up by the swab were eluted as described previously into a pre-weighed microfuge tube. After methanol had evaporated, the tube was weighed again and the mass of silicone calculated. This procedure was replicated 5 times. Approximately 0.15 mg silicone was eluted (mean ± SEM: 0.14±0.04), and this quantity was used for testing of silicone components. To ensure that the measured mass was not derived from the cotton swab itself, 10 unmodified cotton swabs were eluted directly into pre-weighed microfuge tubes and weighed after methanol had evaporated. For each tube, initial mass was identical to mass after methanol had evaporated, confirming that the cotton swab did not contribute to measured silicone mass.

Silicone components were prepared for assays by placing a droplet of each component into a pre-weighed microfuge tube, calculating the mass of the component, dissolving it in 500 µl 100% methanol, then transferring a volume corresponding to 0.15 mg to a glass test tube. For combinations of components, volume was divided accordingly as to have a total of 0.15 mg silicone in the test tube. Methanol containing silicone components was dried completely within a fume hood, and the residual was resuspended directly in assay buffer. Trypsin and transglutaminase assays were conducted as described in the mechanical removal section above.

### Statistical analysis

Statistical analyses were conducted using GraphPad Prism version 5.0, with calculations based on raw absorbance values. For assays with native barnacle glue, data were compared using paired t-tests (two-tailed), because glue from the same barnacle was tested with treatment and control. For assays with purified enzymes statistical analyses were by one-way analysis of variance (ANOVA). If assumptions of normality and equality of variance, tested using the Kolmogorov and Smirnov method and Bartlett's test respectively, were met by log transforming data, parametric ANOVA was used and treatments groups were compared to the control group using a Dunnet's method post-hoc test. If assumptions of normality and equality of variance could not be met after log transforming data, non-parametric Kruskal-Wallis analysis was used and treatment groups were compared to the control group using a Dunn's method post-hoc test. Statistical analyses of enzyme activity with model polysiloxane coating components were conducted separately for components tested individually and when components were tested in combination.

## Results

### Methanol rinses of commercial silicone films: GC-MS

**Figure 1:**
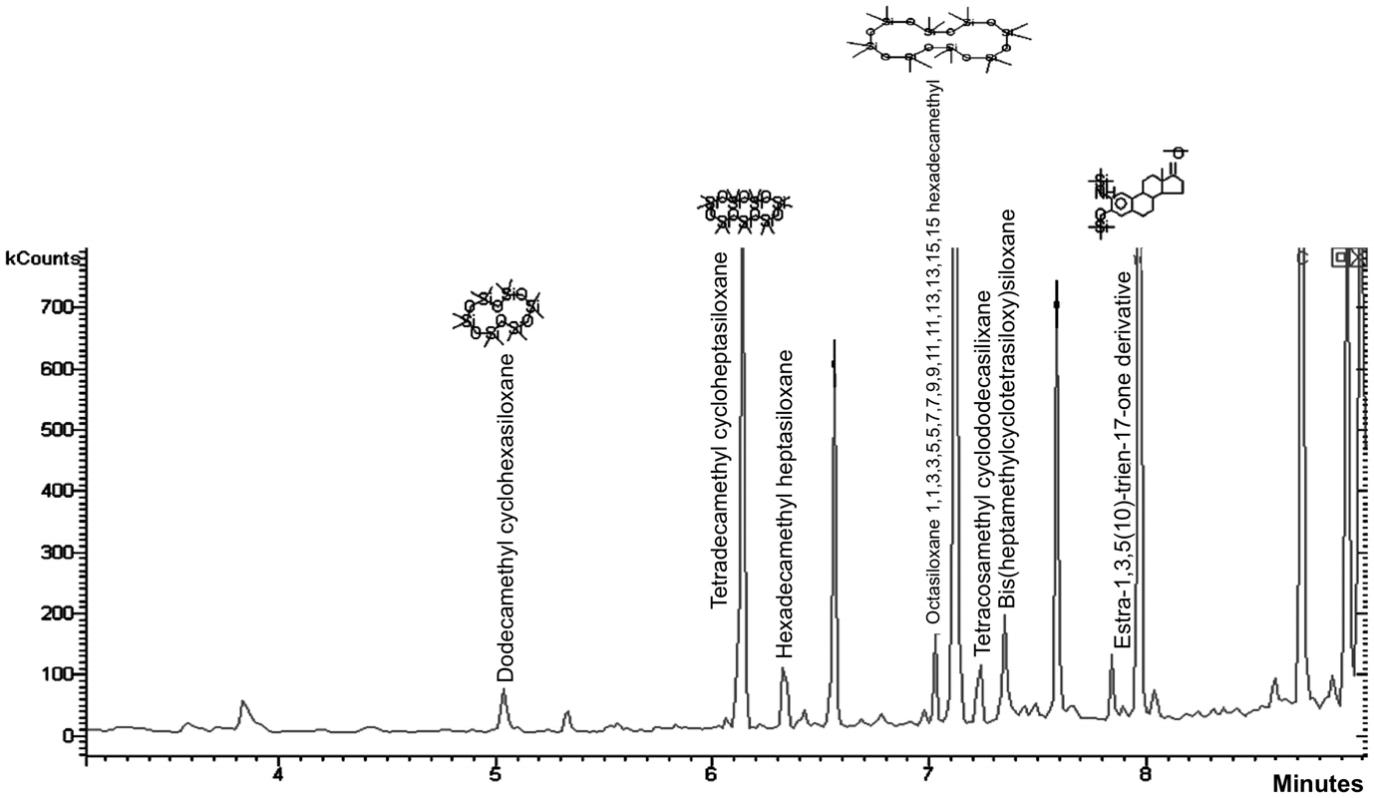
Gas chromatogram and tentative peak assignment (NIST database) for compounds present on Dow Corning Silastic T2^®^ silicone. Samples were obtained by 30 second, 30 µl methanol rinses. Panels had been conditioned in flowing seawater and then used as barnacle growth substrates, immersed in seawater, for an approximate total of 1½ years before use in this analysis.

**Figure 2:**
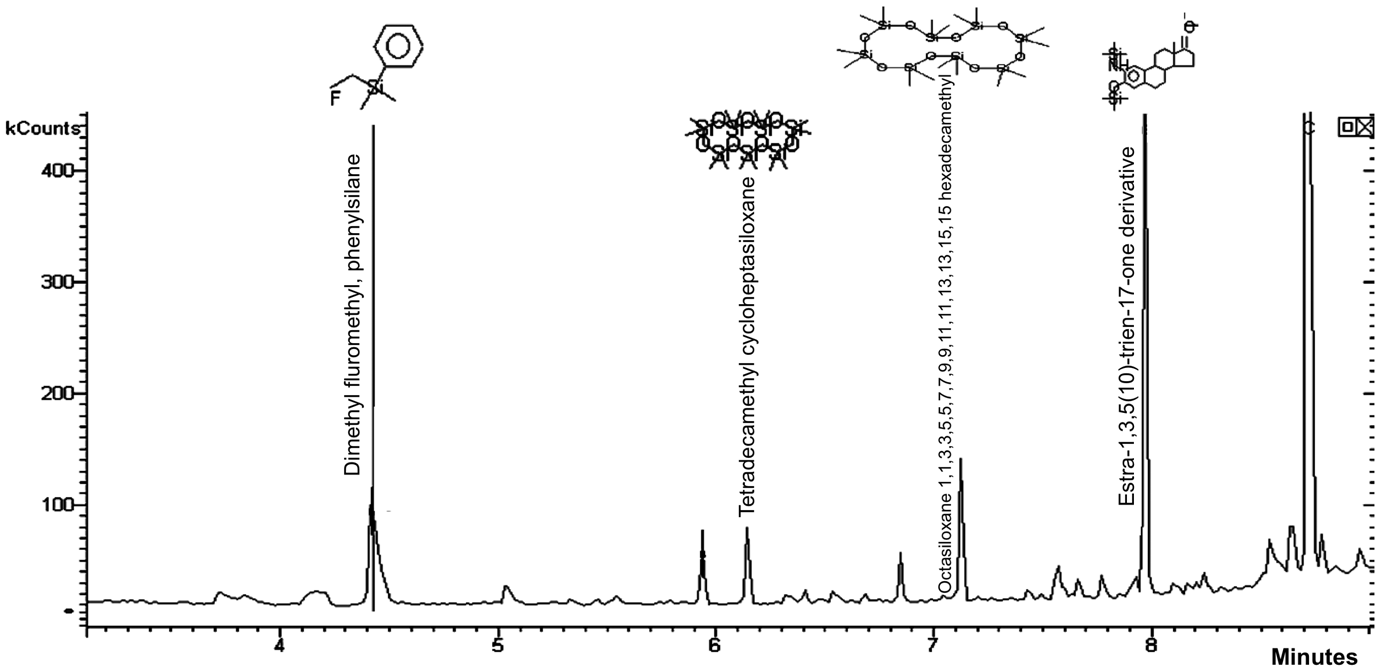
Gas chromatogram and tentative peak assignment (NIST database) for compounds present on International Paints Veridian^®^ silicone. Samples were obtained by 30 second, 30 µl methanol rinses. Panels had been conditioned in flowing seawater and then used as barnacle growth substrate, immersed in seawater for an approximate total of 1½ years before use in this analysis.

We used coupled gas chromatography-mass spectrometry to assess T2^®^ and Veridian^®^ silicones for surface-associated compounds. T2^®^ and Veridian^®^ had been conditioned in flowing seawater until they were not toxic to barnacle larvae, and then immersed in seawater at two month intervals as barnacle growth substrates. Each substrate was exposed to seawater for a total time of approximately 1½ years prior to their use in this study. Thirty second, 30 µl methanol rinses of these silicones contained organosiloxanes and probably cyclic siloxanes. Tentative identifications of compounds are shown in figures 1 and 2. In Silastic T2^®^ rinses, 7 major GC peaks were identified as siloxanes (Figure 1). As shown in Figure 2, 4 major GC peaks were identified as siloxanes for Veridian^®^. With the exception of dimethyl flouromethyl phenylsilane, which might be derived from the catalyst, all compounds that could be identified from Veridian^®^ rinses were also present in T2^®^ rinses. The silicone conjugated Estra-1,3,5(10)-trien-17-one derivative, identified for both T2^®^ and Veridian^®^, was not part of the original coating formulations (Coatings Industry representatives, personal communication).

### Methanol rinses of commercial silicone films: enzyme assays

**Figure 3:**
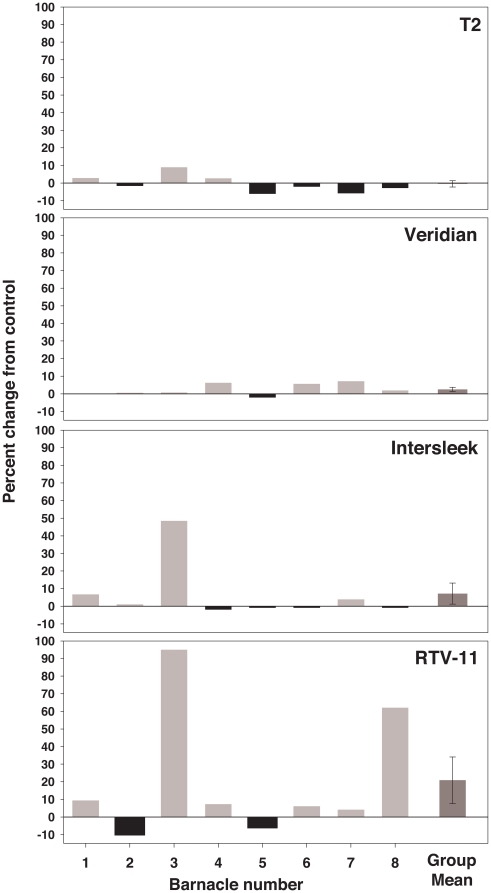
Effect of silicone rinses on barnacle glue trypsin activity. 30 second, 60 µl methanol rinses were conducted, 10 rinses were pooled, dried completely, and residual was resuspended in 10 µl 100% methanol before adding assay buffer. Each individual barnacle was tested with all four silicones. Individual data, expressed as percent change in OD_405_ from control, and group mean (± SEM) are shown. The control is barnacle glue incubated with 10 µl 100% methanol and assay buffer only, without silicone residual.

**Figure 4:**
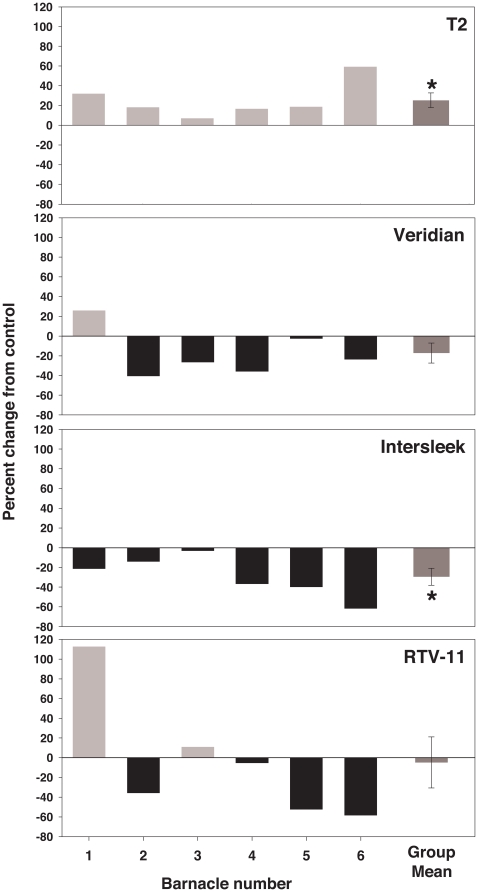
Effect of silicone rinses on barnacle glue transglutaminase activity. 30 second, 60 µl methanol rinses were conducted, 10 rinses were pooled, dried completely, and residual was resuspended in 10 µl 100% methanol before adding assay buffer. Each individual barnacle was tested with all four silicones. Individual data, expressed as percent change in OD_450_ from control, and group mean (± SEM) are shown. The control is barnacle glue incubated with 10 µl 100% methanol and assay buffer only, without silicone residual. * Indicates a significant difference from control (paired t-test: p<0.05).

The effect of the residue of silicone rinses on barnacle glue trypsin and transglutaminase activity is shown in Figures 3 and 4. The impact of residues was dependent on the source of curing glue. HPLC grade methanol control assays showed that neither trypsin nor transglutaminase activity varied significantly in glue assays with methanol versus glue assays with deionized water substituted for methanol (paired t-tests: trypsin: p = 0.162, n = 7 barnacles; transglutaminase: p = 0.161, n = 8 barnacles).

When tested with curing barnacle glue, group mean trypsin activity did not differ significantly from methanol controls for any of the silicones tested (paired t-tests; Figure 3). In contrast, barnacle glue transglutaminase activity differed significantly from methanol controls for T2^®^ and Intersleek^®^ (paired t-tests: p<0.05; Figure 4), with activity dependent upon silicone source. Individuals tested with residues from T2^®^ showed only promotion of activity and those tested with residues from Intersleek^®^ showed only inhibition. Transglutaminase activity was dependent on the individual barnacle producing the glue when tested with Veridian^®^ and RTV-11^®^ residues, resulting in both promotion and inhibition of activity observed.

**Figure 5:**
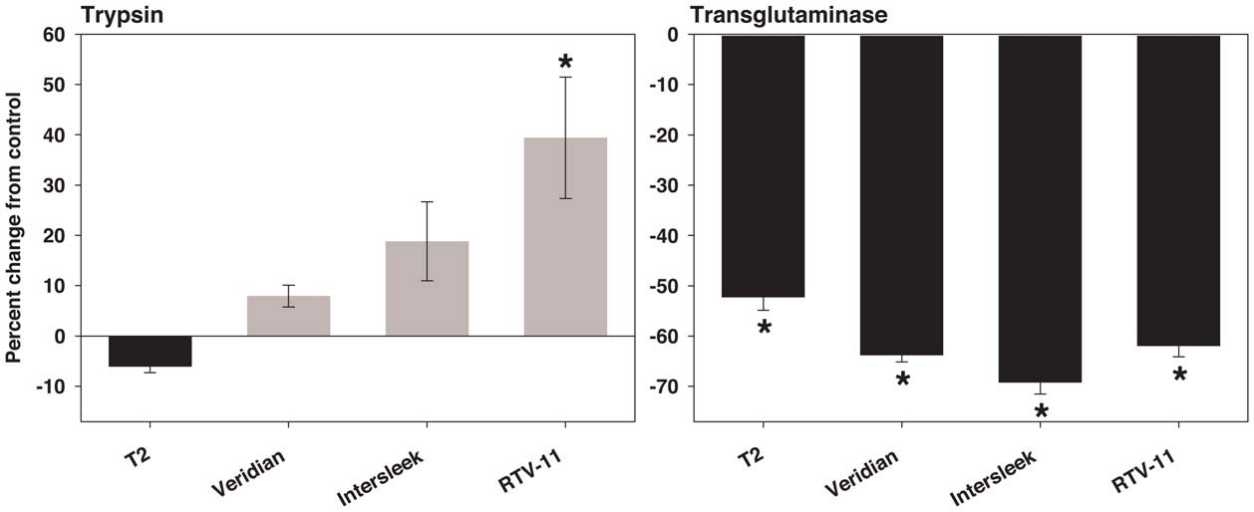
Effect of silicone rinses on purified trypsin and transglutaminase activity (from porcine and guinea pig respectively). 30 second, 60 µl methanol rinses were conducted, 10 rinses were pooled, dried completely, and residual was resuspended in 10 µl 100% methanol before adding assay buffer. Data are expressed as percent change in OD_405_ (trypsin) or OD_450_ (transglutaminase) from control. Means and SEM are shown. The control is purified enzyme incubated with 10 µl 100% methanol and assay buffer only, without silicone residual. * Indicates a significant difference from control (trypsin: Dunn's method post-hoc analysis, p<0.05; transglutaminase: Dunnet's method post-hoc analysis, p<0.05). n = 5 replicates for trypsin, 10 replicates for transglutaminase.

The effect of silicone residues on purified trypsin and transglutaminase is shown in Figure 5. Trypsin activity varied significantly among silicone residue and methanol control groups (Kruskal Wallis One-way ANOVA on ranks: p = 0.0004). The activity of RTV-11^®^ residues differed significantly from the methanol control (Dunn's method post-hoc analysis: p<0.05). Transglutaminase activity varied significantly among silicone residue and methanol controls (One-way ANOVA: p<0.0001). Assumptions of normality and equality of variance were met after log transforming OD_450_ values (normality tested with Kolmogorov and Smirnov method, p>0.05 for each group; equality of variance tested with Bartlett's test, p = 0.4198). Post-hoc analysis showed that all silicone residues inhibited enzymatic activity and each differed significantly from the methanol control (Dunnet's method post-hoc analysis: p<0.05).

### Mechanical removal of compounds on silicone films: GC-MS

The mechanical removal method involved gently rubbing model polysiloxane films with a cotton swab. These 8 films had been conditioned in deionized water for 14 days prior to assays. As detailed in Table 1, multiple poly(dimethylsiloxanes) with similar mass-to-charge fragments were identified for all samples, including dry and methanol wetted swabs. Amino-substituted polysilaxanes were identified only for wetted swab samples of the four surfaces containing silicone oil.

**Table 1:** Gas-chromatographic retention times of peaks with characteristic mass fragments belonging to poly(oligomethylsiloxanes) and amino-substituted polysilaxanes in dry and wet surface swabs obtained from model polysiloxane coatings.

Silicone sample				Retention time (min)										
MW	CL	Oil	Swab	8.05	9.54	9.68	9.77	9.99	10.29	10.98	11.55	11.65	12.03	12.48
L	L	−	Dry		▪				▪	▪		▪	▪	▪
L	H	−			▪				▪	▪		▪	▪	▪
H	L	−			▪				▪	▪		▪	▪	▪
H	H	−			▪				▪	▪		▪	▪	▪
L	L	+			▪				▪	▪		▪	▪	▪
L	H	+			▪				▪	▪		▪	▪	▪
H	L	+			▪				▪	▪		▪	▪	▪
H	H	+			▪				▪	▪		▪		▪
L	L	−	Wet	□	▪				▪	▪		▪	▪	▪
L	H	−		□	▪				▪	▪		▪	▪	▪
H	L	−		□	▪				▪	▪		▪	▪	▪
H	H	−		□	▪				▪	▪		▪	▪	▪
L	L	+		□	▪	○	•	▴	▪	▪	◊	▪	▪	▪
L	H	+		□	▪	○	•	▴	▪	▪	◊	▪	▪	▪
H	L	+		□	▪	○	•	▴	▪	▪	◊	▪	▪	▪
H	H	+		□	▪	○	•	▴	▪	▪	◊	▪	▪	▪

The samples under investigation were characterized by high (H) and low (L) molecular weight (MW), polymerized with the addition of low (L) and high (H) amounts of cross linker (CL), and with (+) or without (−) the addition of silicone oil (Oil). Different organosiloxanes with similar mass-to-charge fragments (73, 147, 221, 281, 355, 429) are denoted by (▪)†. Characteristic mass fragments in different polysilaxanes were □ (351, 379); ○ (87, 115, 351, 379, 437); • (87, 115, 277, 421); ▴ (87, 115, 337, 481); ◊ (87, 115, 439, 583). † As polydimethylsiloxanes of different ring size show almost identical mass fragmentation patterns the exact elucidation of repeat units (*n*) was not possible.

### Mechanical removal of compounds on silicone films: enzyme assays

**Figure 6:**
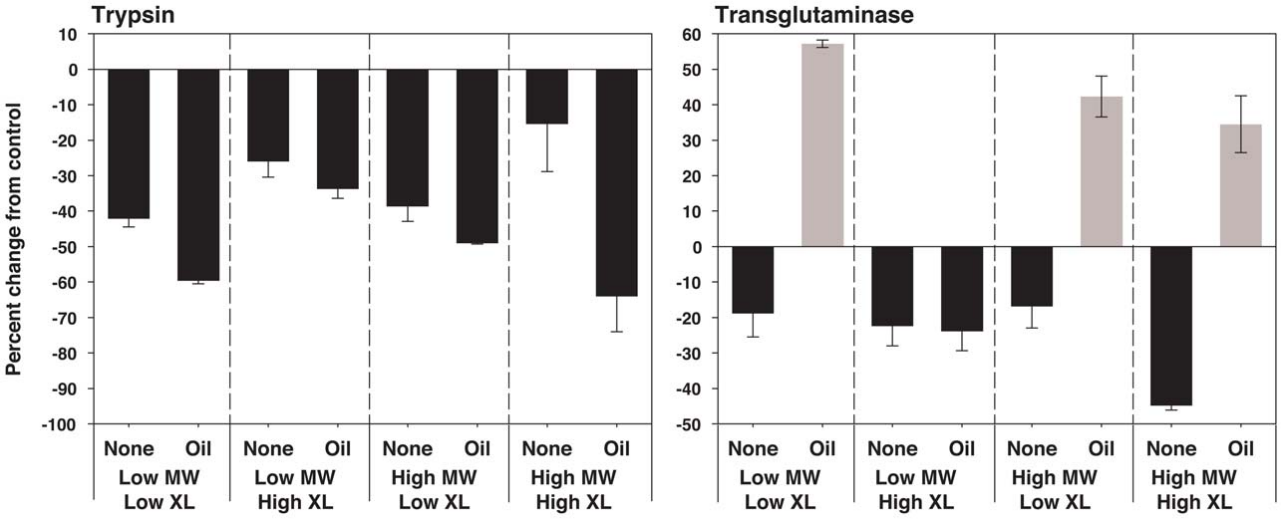
Effect of silicone eluates on purified trypsin and transglutaminase activity (from porcine and guinea pig respectively). Elution was conducted by swabbing model polysiloxane coatings with a dry cotton swab. Compounds taken up onto the cotton swab were eluted with methanol, methanol was then dried completely, and residual was resuspended directly in assay buffer with no additional methanol added. Controls were clean cotton swabs that had not been in contact with polysiloxane, which were eluted, dried, and resuspended in assay buffer. Model polysiloxane coatings were composed of low or high molecular weight (MW) oligomers, and were prepared with high or low concentration of cross-linker (XL), with or without silicone oil. Data are expressed as percent change in OD_405_ (trypsin) or OD_450_ (transglutaminase) from control. Means and SEM are shown. n = 2 replicates.

Eight model polysiloxanes were rubbed with methanol cleaned, dry cotton swabs. Sorped compounds were eluted from swabs with methanol, dried and resuspended in enzyme assay buffer. Controls were methanol cleaned and dried cotton swabs eluted with methanol, dried, and then resuspended in assay buffer. All model polysiloxane coating residues inhibited trypsin activity as compared to controls (Figure 6). In each case, the percent change from control was greater in coatings with silicone oil. Transglutaminase activity was inhibited as compared to controls for each of the silicones that did not contain silicone oil (Figure 6). Three of four model silicones with silicone oil showed a 34 to 57% promotion of transglutaminase activity as compared to controls. Variation between replicates was less than 27% for all enzyme assays.

### Effects of silicone components on enzyme activities

**Figure 7:**
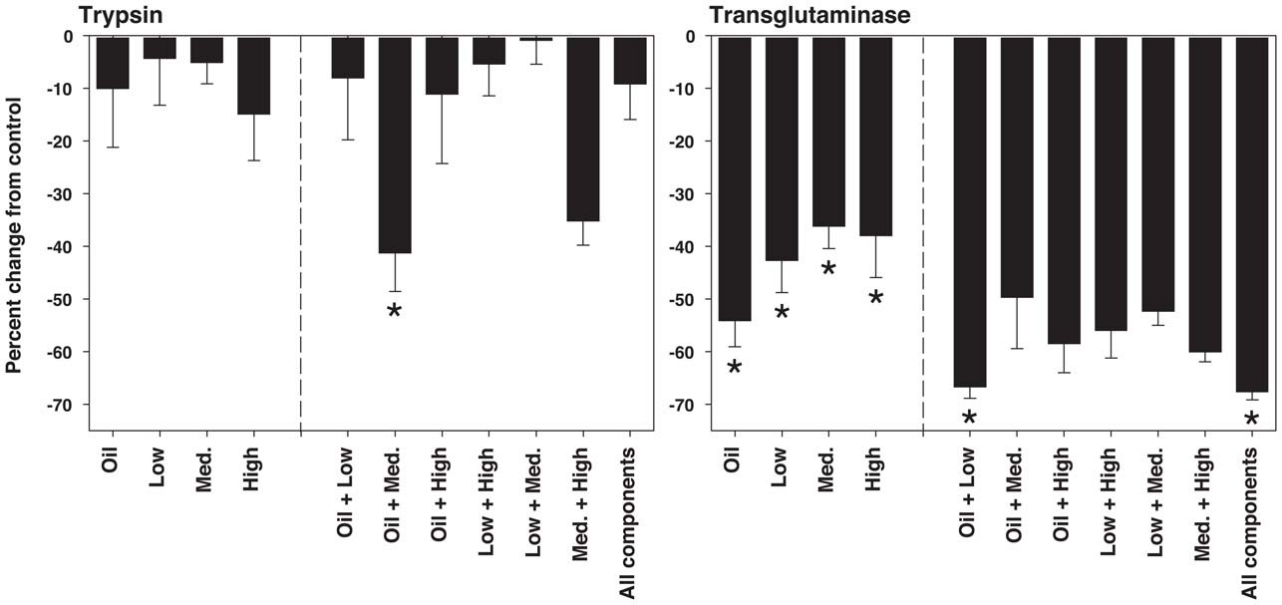
Effect of silicone oil and PDMS oligomers on purified trypsin and transglutaminase activity from porcine and guinea pig, respectively. Silicone oil (viscosity 40–50 cSt) and low, medium and high molecular weight PDMS oligomers (viscosity 700–800, 1000, and 5000 cSt respectively) were tested alone and in combination. Components were dissolved in methanol, the methanol was then dried completely, and residual was resuspended directly in assay buffer. Data are expressed as percent change in OD_405_ (trypsin) or OD_450_ (transglutaminase) from control. Means and SEM are shown. The control is purified enzyme incubated with assay buffer only, without silicone components. * Indicates a significant difference from control (Dunn's method post-hoc analysis: p<0.05). n = 10 replicates for individual components, 5 replicates for combinations.

The components of model polysiloxane coatings that alter enzyme activity were determined using commercial trypsin and transglutaminase (Figure 7). Components were tested individually and in combination, and included silicone oil, and low, medium and high molecular weight PDMS oligomers. The controls for these assays were purified enzymes incubated without silicone components. Non-parametric analyses were employed for these comparisons, since data did not meet assumptions of normality and equality.

When tested with individual silicone components, trypsin activity was not significantly different than controls (Kruskal Wallis One-way ANOVA on ranks). When tested in combination, however, trypsin activity varied significantly among test and control groups (Kruskal Wallis One-way ANOVA on ranks: p = 0.0171). Activity of the oil plus medium molecular weight PDMS (1000 cSt) group differed significantly from that of the control (Dunn's method post-hoc analysis: p<0.05).

Whether tested with individual components or with combinations, transglutaminase activity differed between test and control groups (Kruskal Wallis One-way ANOVA on ranks: p = 0.0005 and p = 0.0018 respectively). Activity with all four components differed significantly from the control when tested individually (Dunn's method post-hoc analysis: p<0.05). The oil plus low molecular weight PDMS and all components combined groups differed significantly from the control (Dunn's method post-hoc analysis: p<0.05).

## Discussion

Weak attachment on silicone foul-release coatings results from a combination of physical and chemical properties of the polymer. This report focused on chemical interactions of silicone polymers with curing glue. We tested the hypothesis that compounds associated with silicone polymer surfaces alter the activity of enzymes involved in barnacle glue curing. Three specific questions were addressed: 1) are compounds available at the surface of silicones; 2) if compounds are available, do they alter barnacle glue enzyme activities, and; 3) which components of silicone polymers alter enzymatic activity. GC-MS identified surface-associated siloxanes on all silicones, including those with long-term exposure to seawater. Surface-associated compounds significantly altered transglutaminase activity in curing barnacle glue. Statistically significant changes in trypsin and, to a greater extent, transglutaminase activity occurred when specific polysiloxane components were tested alone and in combination.

In our glue curing model [15], trypsin activity activates pro-forms of structural proteins, enabling them to rearrange and present domains with compatible motifs [18]–[20], [28] to the surface. Transglutaminase cross-linking locks the polymers in place [15]. Phenyloxidase activity has also been observed and may be involved in glue curing [12] although its function unclear [21]. In this coordinated process, modification of enzymatic rates occurring through specific or non-specific interactions would alter the ability of glue to make adhesive bonds.

GC-MS showed compounds are available at the silicone surface, enabling them to interact with the structural proteins and enzymes in glues. Silicone components are routinely found at the surface of PDMS coatings, and interfere with contact angle measurements [29]. Consistent with this, Meyer et al. [9] showed the presence of surface-active eluates from silicone coatings based on contact angle anomalies. Assays were performed with 12 diagnostic fluids with chemistry mimicking that of amino acids found in bioadhesive proteins. Results of contact angle measurements suggest residues have the potential to alter the curing of biological glues [9]. Our 30-second methanol rinses of commercial polymers exposed to seawater for over a year, showed a variety of siloxanes available at the surface. Similar results were obtained when model polysiloxane coatings were rubbed briefly with a dry cotton swab. GC peaks were assigned primarily to cyclic siloxanes of different ring size. Surface-associated siloxanes could either be unreacted reagents or degradation products of high molecular weight siloxanes.

Silioxanes identified by GC-MS could alter activity of enzymes through specific or non-specific interactions. Some ways silicone oligomers and oils may alter enzyme activity include: interaction with the enzyme active site; encapsulation of the enzyme [30]; altering protein tertiary or quaternary structure by bulk interactions; and by binding of cofactors. When a combination of siloxanes is surface available and exposed to a complex proteinaceous glue, interactions are likely to be complex. Due to the highly coordinated nature of the glue curing process, slight alterations in enzyme activity could have major impacts on curing.

In addition to cyclic siloxanes, GC-MS identified other compounds that might interact with curing glues. For example, GC-MS analysis of commercial polymers revealed the presence of a siliconized estradione, as shown in figures 1 and 2. The estradione-silicone hybrid was not part of the original coating formulations (Coatings Industry representative, personal communication), and was likely derived through microbial metabolism. This result suggests that organisms can partially metabolize surface-available siloxanes and generate novel compounds with unknown bioactivity, stability, fates, and effects. The open electrometric nature of silicone coatings enables uptake of such compounds from the surface or external seawater into the coating [31].

Lightly rubbing silicone surfaces with cotton swabs was a mechanical method to test for compounds available at the silicone surface. The amount of compound was greater than what a barnacle would encounter instantaneously, because the amount represents what would be found in two square centimeters. Quantitative genetics data on glue phenotypes, however, shows that the glues are modified in a silicone polymer dependent fashion that is not based upon physical aspects of the silicones [23], suggesting the concentration of available compounds is sufficient to interact with glue. The types of compounds identified with the mechanical method were similar to those identified from methanol rinses. The actual concentration of compounds that would be available to interact with curing glue under natural conditions is currently unknown. Silicon has been found incorporated into the adhesive plaque of barnacles grown on silicone coatings [7], [8], however, indicating that compounds can partition into and interact with biological glues under environmental conditions. Our transglutaminase assays with uncured glue showed clear interaction with available components. For fouling organisms such as barnacles, the relevant elution solvent is a proteinaceous glue. GC-MS studies using proteinaceous glue as a solvent will shed light on the composition and concentrations of compounds available to interact with curing glues.

The interaction of surface-available compounds with enzymes in curing glue was demonstrated using curing barnacle glue and purified, commercially available forms of the enzymes. The effect of silicone residues on enzymatic activity was less dramatic and consistent when tested with limited quantities of native enzymes than when purified enzymes were used. Trypsin and transglutaminase activity was found in all barnacle glue samples assayed. The lower activity, as compared to that of purified enzymes, resulted in decreased sensitivity and a decreased ability to discriminate between treatments.

Two additional factors contributed to decreased analytical precision with curing barnacle glue. First, mixing with reagents is difficult since curing begins immediately upon release by the barnacle. Second, barnacle glue contains a large number of components [15], [18], [19], [25], [28]. The ideal assay would measure the interaction of glues with the surface as they are released.

Silicone compounds may interact with many glue components including structural proteins, non-proteinaceous components, cofactors, and cells. This complex set of interactions results in variable enzymatic responses. In the presence of silicone residue barnacle glue enzymatic activity varied depending on the individual barnacle, particularly for transglutaminase. This response is consistent with classic studies of barnacle isozymes [32] and with heritable variation in adhesive traits of barnacles raised on silicone coatings [23], [33]. Variability in enzymatic response reflects individual variability in the multicomponent mixture that becomes cured glue.

Although transglutaminase activity levels were low and variable among individuals, silicone residues significantly altered transglutaminase activity in curing barnacle glue. It is noteworthy that for transglutaminase activity, a statistically significant result was shown when inhibiting activity (Intersleek^®^) and promoting activity (T2^®^). We hypothesize this result reflects differences in coating formulations, and the ability of coating components to interact with transglutaminase within a complex proteinaceous environment. Our current model for barnacle glue curing [15] depicts the curing mechanism as a highly interdependent process akin to blood clotting [34]–[37], in which enzymatic activity can affect both up and down stream processes. Hence, significant alteration of enzymatic activity, either inhibition or promotion, has the potential to alter curing and adversely effect adhesive properties, contributing to variable but low adhesion strength of barnacle glue on silicones.

To directly address if silicone coating components alter trypsin and transglutaminase activity, purified enzymes were tested with residues of silicones collected by swabbing silicone surfaces, and with pure polysiloxane coating components. Trypsin activity was inhibited, specifically by silicone oil plus medium molecular weight PDMS and by residue on dry swabs from model silicone polymers. Statistically significant promotion of activity was observed with RTV-11^®^ residue. For transglutaminase, statistically significant inhibition of activity was shown for compounds from all commercial silicones, all polysiloxane components, and specific component combinations. Residues from the surfaces of model polysiloxane coatings containing oil repeatedly promoted transglutaminase activity in all but one polymer tested. This result suggests that the amount and type of surface available compounds differs when they have been consolidated into a polymer versus when they are introduced free into an assay. The chemistry of polymerization produces compounds that are not found in the starting material, which could alter enzyme activity. Knowledge of the direction and magnitude of these enzymatic alterations will enable investigation of their effect on downstream biological processes. Throughout this study, the effects of silicone compounds were shown to be more dramatic and consistent for transglutaminase than for trypsin. The difference in enzyme alteration may be due to the nature of the enzyme itself and its susceptibility to the specific compounds tested, or due to the sensitivity of the enzymatic assays.

### Conclusions

Barnacles exhibit low adhesive strength [38] and frequently produce atypical soft, thick glue when grown on silicone polymers [8], [39]. On silicone, barnacle glue is a hydrated viscoelastic gel, varying with distance to the substrate [40]. Data presented here showed that all four silicone components tested were capable of altering the activity of purified transglutaminase; purified trypsin activity was altered only when medium molecular weight PDMS was combined with silicone oil. Surface available silicone compounds are capable of altering transglutaminase activity in curing glue, as shown for two of four silicones polymers tested. Silicone compounds may also interact with other proteins and non-proteinaceous components of barnacle glue, altering curing. Adhesive strength and glue morphology are complex phenotypes [23], [33]. As for synthetic adhesives, changes in catalytic activity can affect curing, adhesive strength, and morphology of natural glues.

It is common knowledge that after extended exposure to seawater, silicone substrates gradually loose their foul-release properties. Adhesive strength increases and the proportion of barnacles producing thick, gummy glue, a heritable trait [23] is low as compared to newly polymerized silicones (Orihuela, personal observation). Within the context of physical and chemical changes that occur as polymers age, diminished foul-release properties could be partially due to a reduction in the levels and types of silicone surface-associated compounds. This reduction would in turn decrease interference with glue curing. Foul-release properties may be lost as surface-associated diffusible components fall below a threshold level.

The interaction of compounds available at the surface of silicone polymers with curing glues is one of many mechanisms that make silicone foul-release coatings effective. Compounds that comprise silicone polymers and are surface-available alter two pervasive and biologically important enzymes: trypsin-like serine protease and transglutaminase. Work is currently ongoing to determine how broadly trypsin and transglutaminase are employed in marine biological adhesion. The role of exoproteases in the growth of bacterial biofilms is already well established [41]–[43]. The mechanisms described here can potentially be employed in fouling control measures. It would be prudent to investigate the impacts of silicone oligomers and oils on other biological [44] and environmental [45] processes.
